# Intracellular VHHs to monitor and modulate GPCR signaling

**DOI:** 10.3389/fendo.2022.1048601

**Published:** 2022-11-16

**Authors:** Pauline Raynaud, Camille Gauthier, Vinesh Jugnarain, Frédéric Jean-Alphonse, Eric Reiter, Gilles Bruneau, Pascale Crépieux

**Affiliations:** ^1^ Physiologie de la Reproduction et des Comportements (PRC), Institut National de Recherche pour l’Agriculture, l’Alimentation et l’Environnement (INRAE), Centre National de la Recherche Scientifique (CNRS), Institut Français du Cheval et de l’Equitation (IFCE), Université de Tours, Nouzilly, France; ^2^ Inria, Inria Saclay-Ile-de-France, Palaiseau, France

**Keywords:** G protein-coupled receptor, conformations, intracellular VHHs, Cell signaling, G proteins, β-arrestins, biosensor

## Abstract

Single-domain antibody fragments, also known as VHHs or nanobodies, have opened promising avenues in therapeutics and in exploration of intracellular processes. Because of their unique structural properties, they can reach cryptic regions in their cognate antigen. Intracellular VHHs/antibodies primarily directed against cytosolic proteins or transcription factors have been described. In contrast, few of them target membrane proteins and even less recognize G protein-coupled receptors. These receptors are major therapeutic targets, which reflects their involvement in a plethora of physiological responses. Hence, they elicit a tremendous interest in the scientific community and in the industry. Comprehension of their pharmacology has been obscured by their conformational complexity, that has precluded deciphering their structural properties until the early 2010’s. To that respect, intracellular VHHs have been instrumental in stabilizing G protein-coupled receptors in active conformations in order to solve their structure, possibly bound to their primary transducers, G proteins or β-arrestins. In contrast, the modulatory properties of VHHs recognizing the intracellular regions of G protein-coupled receptors on the induced signaling network have been poorly studied. In this review, we will present the advances that the intracellular VHHs have permitted in the field of GPCR signaling and trafficking. We will also discuss the methodological hurdles that linger the discovery of modulatory intracellular VHHs directed against GPCRs, as well as the opportunities they open in drug discovery.

## Introduction

Antibody fragments are currently revolutionizing the specific targeting of molecules inside the cell. Among those, paramount importance has been given to variable fragments from single-chain antibodies encountered in the blood of some Camelidae and cartilaginous fishes. These fragments represent the VHH (VH of heavy-chain only antibodies), and their recombinant analogues have been baptized as Nbs for nanobodies, or sdAbs for single domain antibodies. When compared to conventional immunoglobulins (Igs), they have smaller molecular weight (∼13 kDa) and size (diameter of 2.5 nm, length of 4 nm), and they consist in the minimum paratope able to recognize an epitope. They are composed of 4 framework regions (FR) and 3 complementarity-determining regions (CDR) that support complementarity to the epitope. A striking feature of these VHHs is the greater length of their CDR3 compared to that of a VH, that optimizes the contact surface with the epitope and compensates the lack of light chain. The length and flexibility of CDR3 are properties that are advantageous to reach poorly accessible, cryptic epitopes (1).

VHHs contain a canonical disulfide bond linking FR1 and FR3, and possibly additional bonds, for example between CDR2 and CDR3 in lama. These disulfide bonds are essential for thermal and thermodynamic stability (2), but apparently, reducing conditions do not compromise antigen binding affinity, nor mechanical stability (3). In addition, because of “hallmark” hydrophilic residues at solvent-exposed positions, they are less prone to protein aggregation than single-chain variable fragments (scFv) (4). These properties are of uttermost importance when intracellularly expressed VHHs (intra-VHHs) are being studied, because they are placed in a mildly reducing environment in the cytosol. Although we are aware that the terminology of “nanobody” given by Ablynx in 2001 is widely accepted, in the case of intracellular Nb we prefer the term “intra-VHH” over “intrabodies”, that does not explicitly indicate the format of the antibody (VHH, scFv, Ig). This is the term that will be used in this review.

Intra-VHHs are a new type of biological tools that target proteins present at different sub-cellular compartments [reviewed in (5)], such as the GTP-bound form of RhoA at the inner face of the plasma membrane (6), or lamin lining the nuclear envelope (7). They can be used to enhance, block or monitor (8) protein activation/activity, to track protein trafficking inside the cell when fused to fluorescent probes (7), to hinder protein/protein interactions, or to direct the antigen protein to degradation pathways (9). Some of them appear exquisitely sensitive to protein conformational dynamics, and this chaperone-like property has been particularly useful to decipher the conformational complexity of G protein-coupled receptors (GPCR) in their ligand-activated states.

GPCRs are involved in most physiological responses and as such, they bind ligands encompassing a vast array of biochemical classes, ranging from photons or ions to large glycoprotein hormones. Accordingly, they are major targets of the therapeutic arsenal, including treatments of hypertension, migraine, cancers, metabolic diseases, or disorders of the central nervous system, among other pathologies. But drug discovery of small chemicals with high potency, efficacy and selectivity is reaching a limit, and in 2017, the 481 referenced drugs against GPCRs only targeted 107 out of 850 receptors of this protein family (10). Conventional antibodies now open new avenues in the field, but still, GPCRs remain extremely difficult targets since i/they are highly hydrophobic in nature because of their seven transmembrane helices, ii/most of them expose very limited regions outside the cell membrane, iii/their wide conservation across the animal kingdom goes together with a low immunogenicity, which precludes a strong immune response in heterologous animals. Until now, only two therapeutic monoclonal antibodies that recognize extracellular regions of GPCRs are on the market: mogamulizumab, an anti-neoplasic Ig directed against C-C chemokine receptor type 4 (CCR4) in T cell leukemia (11) and erenumAb, an antagonist of the Calcitonin gene-related peptide type 1 receptor (CALCRL) used to treat migraine (12). The remarkable properties of VHHs now raise exciting prospects for future therapies, because they could stimulate GPCR activity *in vivo* by binding their orthosteric ligand-binding site, or they could modulate the activity of the endogenous ligand by binding to allosteric sites of the receptor. But this line of research is still in its infancy and so far, only the apelin receptor (APLNR) (13) and the metabotropic glutamate receptor 2 (GRM2) (14) have been targeted with agonistic/positive allosteric modulator VHHs, while others, such as CXCR4 (15), are recognized by antagonists. Interestingly, a VHH directed against the parathyroid hormone receptor (PTHR1) has recently been used as a tether of PTH(1–34), a bioactive N-terminal fragment of PTH, in order to improve its selectivity for the receptor (16).

In contrast, the use of intra-VHHs has been essentially limited to academic research. Remarkably, they have been instrumental in overcoming the major challenges associated to the conformational complexity of GPCR in their ligand-activated states. These receptors basally exist in transient conformational states due to rearrangements at the atomic level and at larger receptor portions (17). The corollary of GPCR intrinsic instability is that obtaining the necessary periodic organization required for resolving the 3D structure of ligand-bound GPCRs at high resolution has been a major stumbling block in the GPCR research field.

**Figure 1:**
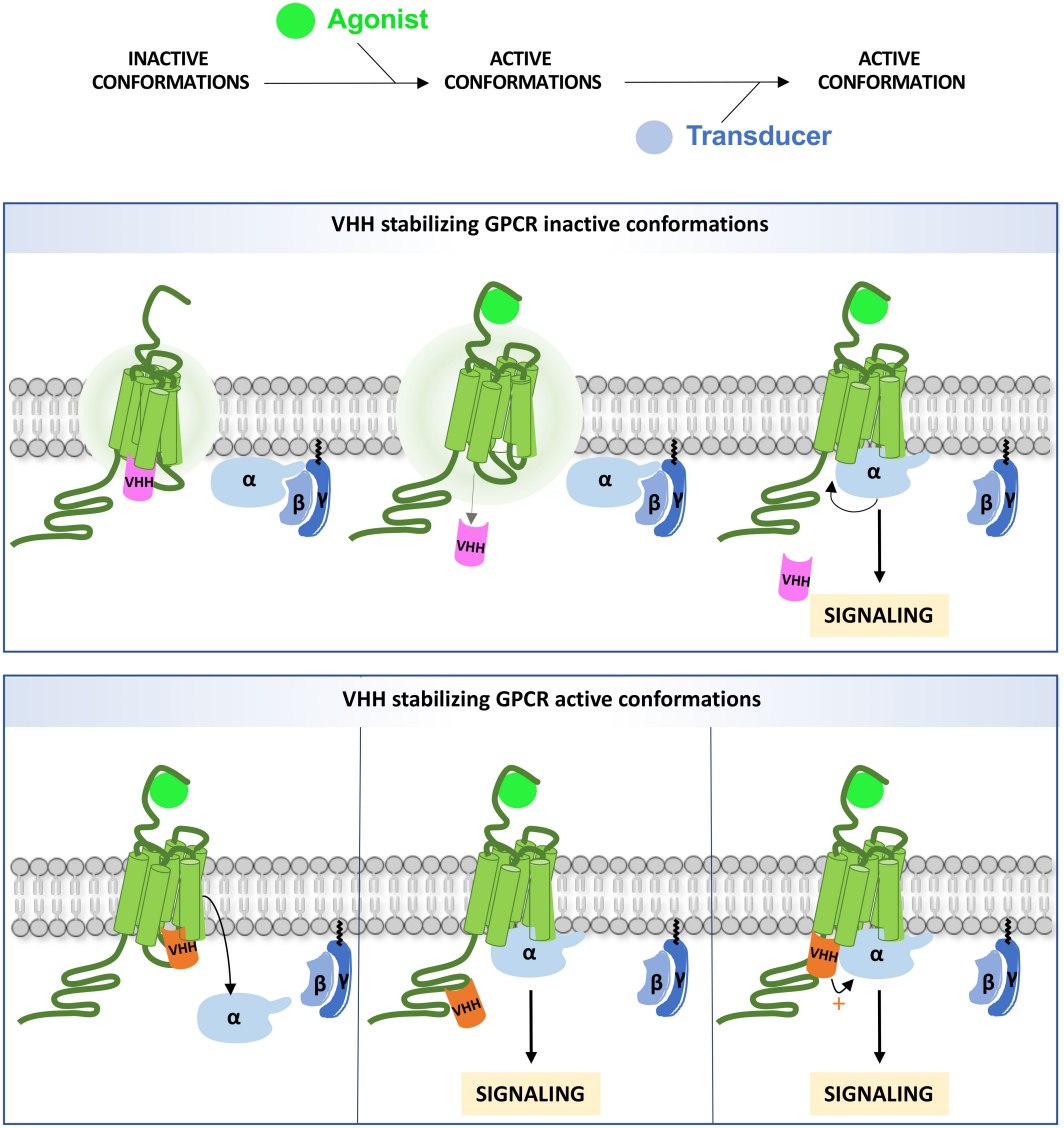
Intra-VHH interference on GPCR signaling. An intra-VHH may stabilize inactive conformations of the GPCR (upper panel) and dissociate upon stimulation. The size of the aura indicates the degree of intrinsic instability. Intra-VHHs may also stabilize active conformations (bottom panel), and compete with G proteins and/or β-arrestins (not shown on the figure) for binding to the receptor, depending on their affinity (left). Alternatively, intra-VHH and transducer binding may be compatible. In that case, GRK phosphorylation sites may be masked (middle) or the VHH may allosterically enhance Gα binding (right).

**Figure 2:**
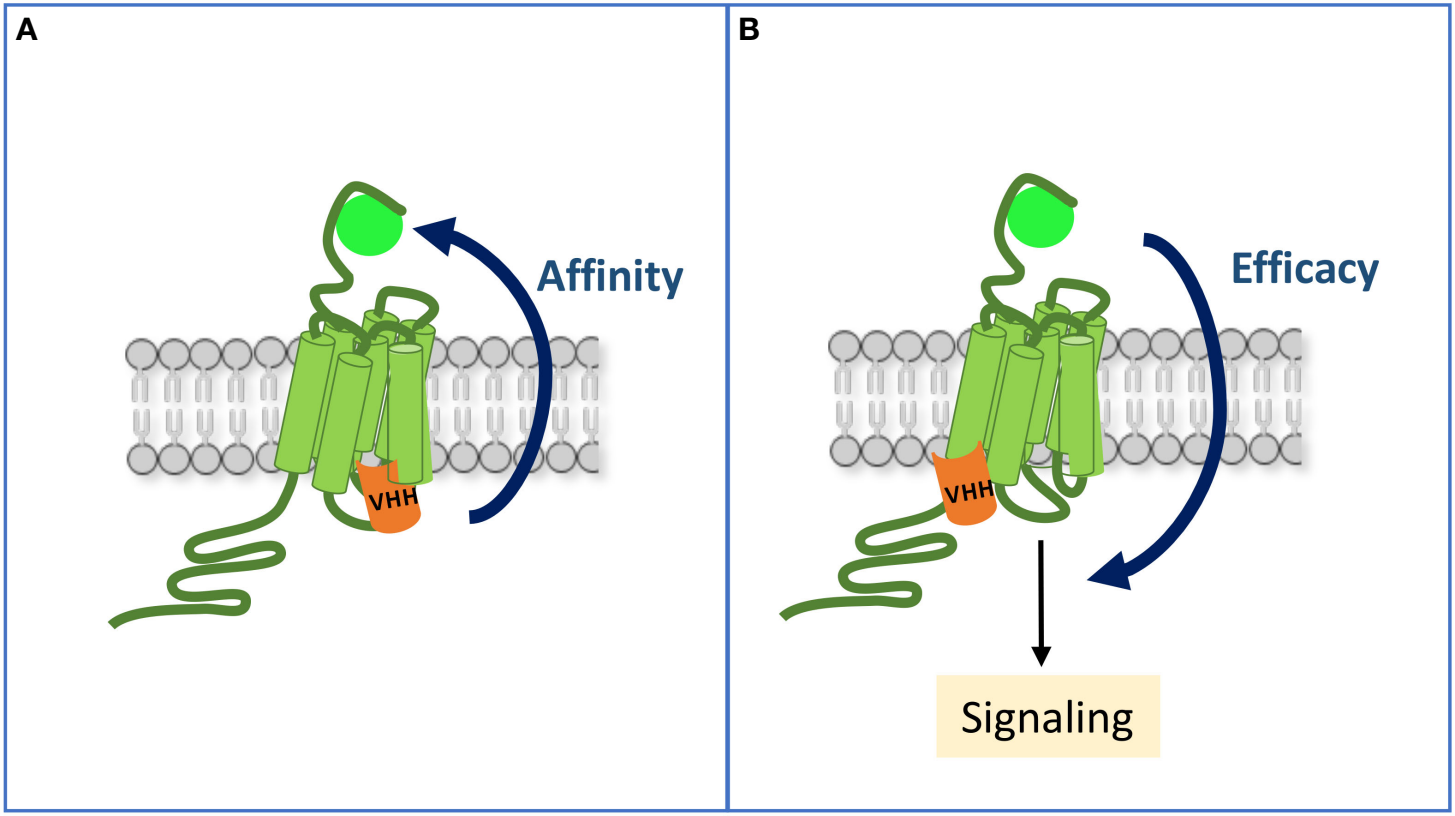
Allosteric communication between the ligand-binding pocket and the intracellular sites of an activated GPCR. **(A)** By mimicking occupancy of the receptor by a transducer, an intra-VHH increases its affinity for the ligand. **(B)** A VHH may also allosterically enhance the efficacy of the ligand to stimulate some signaling pathways, hence inducing a biased response when compared to the reference ligand alone.

These transient conformational states are stabilized by ligand binding and by the subsequent coupling of their intracellular transducing partners, namely G proteins and β-arrestins (**Figure 1**). Hence, there is an allosteric communication whereby ligand binding induces conformational changes at the ligand-binding pocket that propagate to the inner side of the receptor. Allosteric communication goes also the other way round, since, reciprocally, transducer binding enhances the affinity of the receptor for the ligand (18, 19) (**Figure 2**). A decisive breakthrough has been achieved on the G protein and ligand binding pocket allosteric dialog (20–22), with the use of Nb80. Indeed, binding of this antibody to the β2-adrenergic receptor (ADRB2) induces a closed conformation of the orthosteric site that traps the agonist while decreasing its dissociation rate (20, 22). Reciprocal, allosteric coupling of the two regions is essential for full receptor activation. It explains why the crystal structure of ADRB2 bound to a potent agonist unexpectedly matched with the inactive crystal structure of the receptor (23), unless it was stabilized intracellularly (24).

Structural studies have provided detailed insights into the critical epitopes on GPCRs that could be targeted by intra-VHHs in order to be able to lock the receptors in the active, intermediate, or inactive conformations. For class A GPCRs, displacement of the transmembrane domains (TM), in particular TM6, results into a range of ‘open-active’ or ‘close-inactive’ states on the intracellular side of the GPCR, which in turn determines the degree of activity of the receptor, *via* G protein or β-arrestin coupling (25, 26). For instance, the structure of ADRB2 stabilized in the active conformation by Nb80 showed that this intra-VHH possesses G protein mimetic properties (24). Upon agonist binding, Nb80 was capable of maintaining the receptor-activated form by having its CDR3 inserted in the hydrophobic allosteric cytoplasmic pocket, formed by TM segments 3, 5, 6 and 7, otherwise occupied by the C-terminal α-helix of Gαs (27). Leading to major outward displacement of the TM6, Nb80 disrupts the critical ‘ionic lock’ interaction between R131^3.50^ of TM3 and E268^6.30^ of TM6, which would otherwise maintain the receptor in the inactive state (24). Similar G protein mimetic intra-VHHs targeting corresponding regions of muscarinic acetylcholine receptor M1 (CHRM1) (28) and mu-type opioid receptor (OPRM1) (29) have also been structurally described. Partial agonism was demonstrated with intra-VHH Nb71 bound to ADRB2, of which the TM6 had lesser outward displacement (30). Alternatively, acting as a negative allosteric modulator (NAM), intra-VHH Nb60 also had its CDR3 inserted in the allosteric cytoplasmic pocket of ADRB2 (31). However, the inactive state of the receptor was maintained since residues of the CDR3 of the intra-VHH bridged ‘the ionic lock’.

Since 2011, more than a hundred structures of active GPCR conformationally stabilized with intra-VHHs directly or *via* stabilization of G proteins, have been solved. By stabilizing discrete receptor conformational states, in the close future, these single domain antibodies are expected to provide significant insights onto the dynamics of the signaling network and integrated cell response induced by G protein and β-arrestin binding to a ligand- activated GPCR. However, very few VHHs directed against the intracellular parts of GPCRs have been extensively studied functionally. Here, we highlight the outcomes of intra-VHH binding to GPCR intra-cellular regions, or to their transducers, on the receptor signaling properties and trafficking. We will also discuss the methodological pitfalls that preclude extensive studies on GPCR modulation by intra-VHHs, and conclude with the therapeutical avenues that they open in drug discovery.

## Expected insights onto GPCR activity and regulation

Upon agonist binding, GPCRs undergo an ensemble of conformational state transitions enabling the recruitment of G proteins and β-arrestins, that propagate the membrane signal to a complex intracellular signaling network. G protein-coupled receptor kinases (GRKs) also are direct binding partners that desensitize the receptor by phosphorylation and promote β-arrestin-dependent G protein uncoupling and internalization. In addition, GPCRs make direct interactions with less conventional partners, such as HOMER protein homologs (32), PDZ domain-containing protein GIPC (33), Na(+)/H(+) exchange regulatory cofactor NHERF1 (34), vesicle-fusing protein NSF (35) or Multiple PDZ domain protein (MPDZ) (36), that also deserve interest since they generally intervene in receptor targeting to specific subcellular compartments and cell signaling (37). Like G proteins, GRKs and β-arrestins, their interaction with GPCRs could also be modulated by intra-VHHs.

Since anti-GPCR intra-VHHs stabilize selective conformations of the receptor, they have been named “confobodies”, a registered trademark of Confo Therapeutics (38). They can recognize intracellular regions of the receptor devoted to signal transduction (**Figure 1**). Hence, their expected modulatory effect is a competition with direct transducers such as G proteins and β-arrestins by steric hindrance. Alternatively, they may also mask regulatory GRK phosphorylation sites on the receptor. In addition, some intra-VHHs may bind to an active conformation of the receptor without competing with the transducers, by positive allostery. So far, no intra-VHH enhancing the affinity of a transducer for a GPCR has been identified, nor, to our knowledge, intra-VHH forcing a new receptor conformation by induced fit.

## Intra-VHHs to modulate GPCR activity

**Figure 3:**
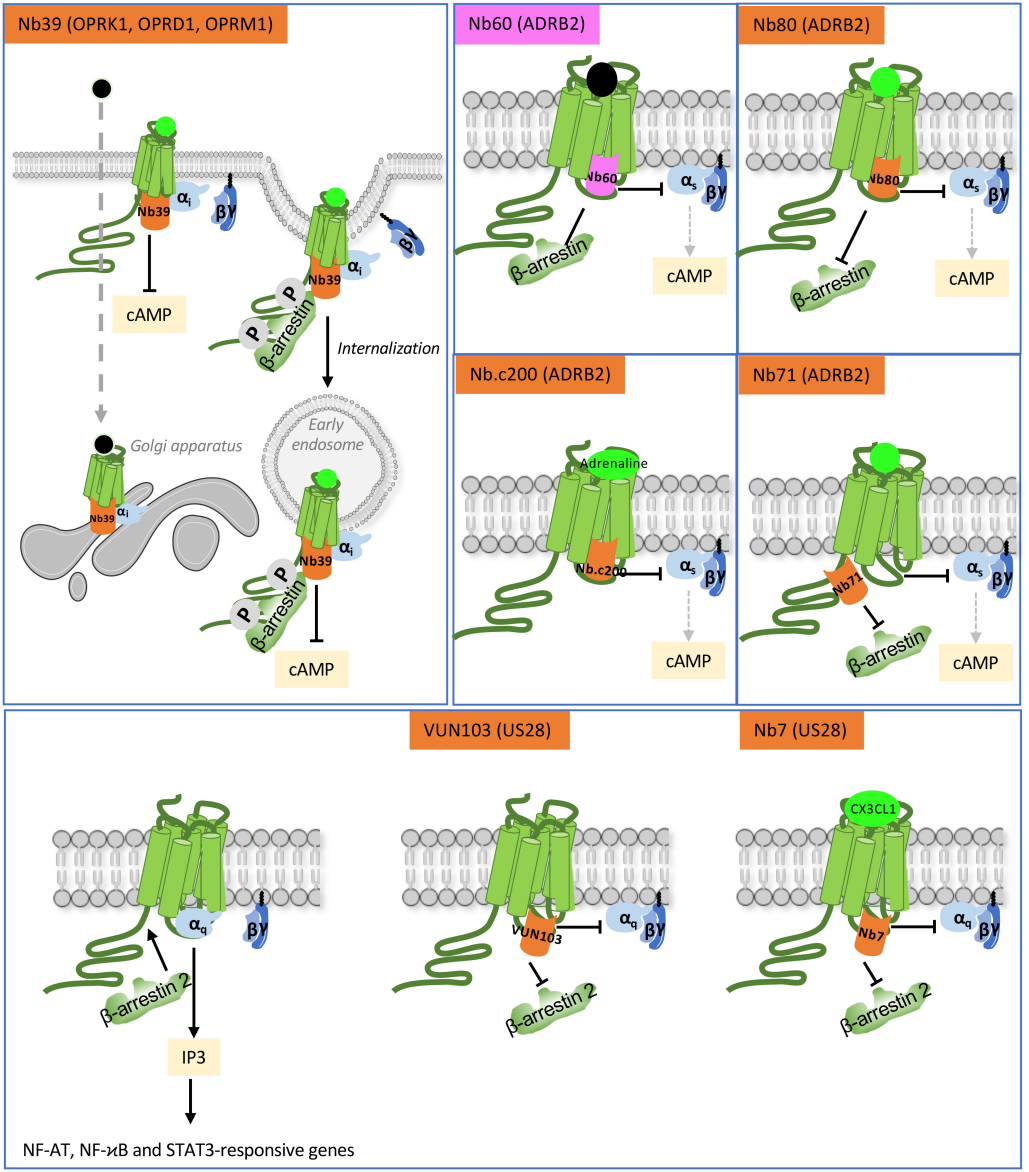
Functional action of intra-VHHs against OPRK1, OPRD1 and OPRK1 opioid receptors, ADRB2 and US28. Nb39 reveals the location of opioid receptors when stimulated by a peptide ligand (bright green) or a cell-permeant non-peptide ligand (black dot). Nb60 locks ADRB2 in an inactive conformation that precludes interaction with transducers. Nb80 stabilizes conformationally active ADRB2. Nb.c200 stabilizes ADRB2 even when stimulated by its natural, low-affinity agonist, adrenaline. Nb71 stabilizes active conformations of ADRB2 and prevents β-arrestin recruitment by limiting access to GRKs. US28 is a constitutively active GPCR. The VUN103 and Nb7 intra-VHH discriminate respectively between constitutively active and agonist-stimulated conformations of the receptor, because Nb7 has no affinity for the apo-conformation. Competition between VUN103 and Nb7 and the transducers hampers US28 signaling and expression of its target genes. Agonists are in bright green, antagonist are in light grey.

The Nb80 intra-VHH and Gαs binding regions on ADRB2 overlap, which explains that the conformation of ADRB2 stabilized by Nb80 or by the Gαβγ trimer are structurally very close (24, 27). As such, this intra-VHH displaces Gαs and stabilizes active conformations of ADRB2 (27). Eighteen other anti- ADRB2 intra-VHHs have been selected from llama immunization with ADRB2 embedded in lipids and covalently bound to the BI-167107 high affinity agonist. These VHHs have been functionally characterized and most of them decreased or inhibited isoproterenol-induced cAMP production and β-arrestin recruitment (39). However, their inhibitory effect likely results from different causes, since 12 of them exhibited preference for agonist-bound ADRB2, which suggests a probable steric hindrance of transducer binding, while the 6 remaining ones, including Nb60, selectively bound to inactive conformations immobilized in the presence of antagonists that do not support transducer recruitment (39). The Nb71 intra-VHH preferentially binds to Isoproterenol-activated receptor, and decreased cAMP production. However, it impaired phosphorylation of ADRB2 by GRKs, hence precluding β-arrestin recruitment and proper receptor intracellular trafficking, which suggests that Nb71 recognizes the carboxy-terminal region of ADRB2 and not the Gs binding region as Nb80 for example, although both intra-VHHs functionally inhibit cAMP production. Four additional anti- ADRB2 intra-VHHs with even better affinity than Nb80 have been isolated from a yeast synthetic library by combining magnetic-bead and fluorescence-activated cell sorting (FACS) enrichments, and they all inhibited the activity of a cAMP-responsive reporter gene, in response to adrenaline, up to 45% of Emax (see Nb.c200 in **Figure 3**) (40).

Biological characterization of intra-VHH is easier for GPCR with diversified ligands and well-described pharmacology. By using agonists and antagonists of a given receptor, it is possible to conclude whether a VHH preferentially stabilizes inactive forms of the receptor or if it binds constitutively the active receptor and is displaced by further recruitment of G proteins, or whether it only binds activated conformations and then prevents G protein binding, provided that it has a sufficient affinity for the receptor, as seems to be the case of Nb80 (estimated Kd ≃140 nM) (41). Several authors have developed a bioluminescence resonance energy transfer (BRET) assay to monitor the interaction of the intra-VHH and a GPCR (42–44). This is the case of the interaction of the kappa-type opioid receptor (OPRK1) with the Nb6 and Nb39 intra-VHHs (**Figure 3**) (42, 45). The profile of dose-response curves obtained in the presence of the salvinorin A (SalA) agonist indicates that Nb6 binds preferentially to inactive OPRK1, whereas Nb39 binds to the receptor only when fully active, and, as expected, enhances SalA binding affinity (42). Accordingly, increasing concentrations of the JDTIc antagonist prevent Nb39 binding to OPRK1 but has no effect on Nb6 binding that recognizes inactive conformations. Interestingly, domain swapping experiments grafting the OPRK1 intracellular loop (ICL3) 3 to seven heterologous receptors physiologically coupled to Gs, Gi or Gq, confer not only Gq recruitment ability but also antagonistic Nb6 binding (45), suggesting that this system can be used to monitor the activation state of Gq-coupled GPCRs. Thus, the Nb6 intra-VHH could be used similarly as the mini-Gq biosensor, and may possibly help de-orphanising orphan Gq-coupled GPCRs (45).

The human cytomegalovirus US28 receptor is an interesting example of a receptor exhibiting high constitutive activity. The latter is due to structural instability of the inactive state, and is only modestly enhanced by ligand binding (46). This observation suggests that constitutively active US28 and ligand-bound receptor have distinct conformations. In agreement, the conformation-specific VUN103 intra-VHH competes with Gq and β-arrestin 2 that constitutively interact with US28, and severely impairs inositol phosphate accumulation, nuclear factor of activated T cells (NFAT), signal transducer and activator of transcription 3 (STAT3) and NF-ϰB activation. In contrast, Nb7 does not affect constitutively active US28, but rather stabilizes US28 bound to its fractalkine CX3CL1 ligand, and inhibits the cell signaling it mediates (44) (**Figure 3**). Although both Nb7 and VUN103 bind ICL2 and ICL3 (46), they seem to recognize distinct active conformations of US28. Importantly, by interfering with US28 constitutive activity, VUN103 partially inhibited the growth and US28-dependent signaling of glioblastoma spheroids as well as glioblastoma cells.

Most G protein mimics described above have affinity for an agonist-bound GPCR, but none of them is able to allosterically enhance receptor-Gα interaction. By using viral evolution of genetically actuating sequences (VEGAS), intra-VHHs against D(2) dopamine receptor (DRD2), the pH-sensing GPR68 and the 5-hydroxytryptamine receptor 2A (HTR2A) receptor have been obtained (43). One of these, VGS-Nb2, was qualified as positive allosteric modulator (PAM). It directly associates to HTR2A and stimulates a serum-responsive element (SRE) responsive reporter gene in the absence of ligand and regardless of Gαq expression, hence inducing an active conformation of the receptor. This response was still enhanced after stimulation by 5HT *via* Gq, coupling, but not β-arrestin 2, whose recruitment to the receptor was decreased. Hence, intra-VHHs isolated by VEGAS are able of inducing receptor activity in the absence of ligand.

## Intra-VHHs as conformational biosensors to explore subcellularly localized GPCR signaling

GPCR intrinsic instability is the major driver of biased signaling. The latter is determined by the selection of discrete conformational states, stabilized by a given ligand, that will ultimately lead to the recruitment of different transducers (*e.g.* G proteins and β-arrestins) with various efficacy or kinetics, according to the ligand (47–50). The high conformational dynamics and heterogeneity of GRKs and β-arrestins and their multiple modes of interactions with GPCR contribute to this diversity (51). The ability of a ligand to selectively stimulate a signaling pathway over the other, when compared to a reference ligand, has been mainly approached as an indirect measure of G protein vs β-arrestin-dependent downstream signaling, and estimated by calculation with the operational model and variants thereof (52–54). But it is not directly informative of receptor conformation. By providing important advance onto active receptor trafficking, intra-VHH conformational biosensors provide insights directly at the level of the receptor on the mechanisms underlying signaling bias.

**Figure 4:**
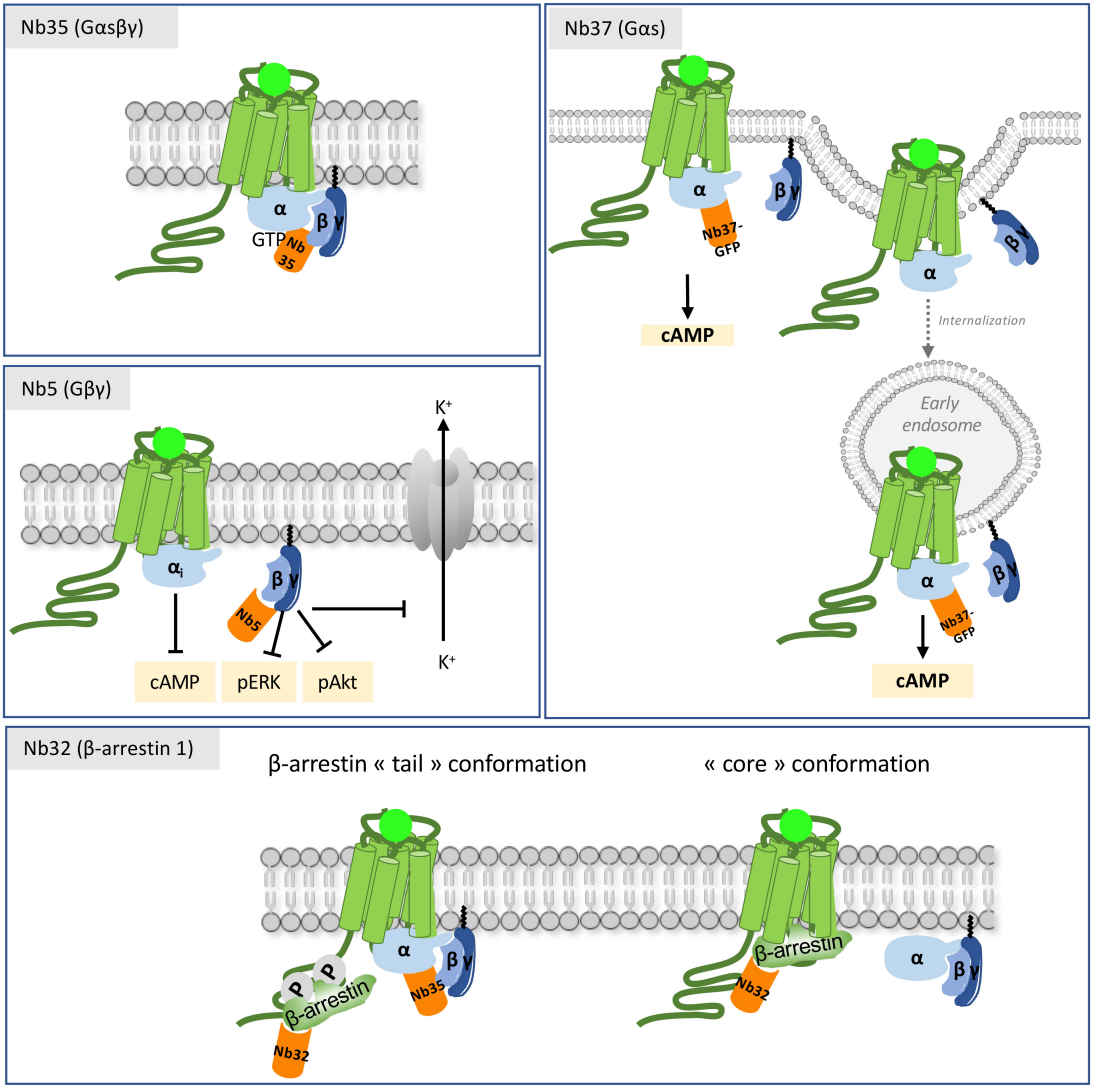
Mechanisms of action of intra-VHHs against GPCR transducers. Nb37 recognizes the nucleotide-free form of active Gαs, and has been used as a conformational biosensor to track receptor activation and signaling throughout the cell compartments. Nb35 stabilizes the interface between Gα and Gβγ, and prevents GTP release. Nb5 binds to Gβγ. It has a pronounced effect on Gβγ signaling (adenylate cyclase, pERK and pAkt) and on the K^+^ current of the GIRK channel in the striatum. Nb32 is an anti-β-arrestin 1 VHH. Nb35 and Nb32 have been used to reconstruct the structural arrangement of the megacomplex composed of an agonist-stimulated ADRB2-V2R chimera simultaneously bound to Gα and to β-arrestin 1.

GPCR signaling and trafficking are closely connected, since it has been unambiguously demonstrated that GPCR signaling does not exclusively take place at the plasma membrane, but also occurs in several intracellular compartments, ultimately leading to distinct biological outcomes (55–59). This conceptual advance has been enabled by repurposing intra-VHHs sensitive to GPCR conformational dynamics initially developed for structural biology. These antibodies can be converted to genetically-encoded conformational biosensors by fusion to a fluorescent protein derived from green-fluorescent protein (GFP). With these tools, it becomes possible to detect where activated receptors are located inside the cell. The first anti-GPCR intra-VHH biosensor was derived from Nb80, that contributed to the resolution of activated ADRB2 3D structure (24). By tracking isoproterenol-activated ADRB2 inside the cell, this biosensor has uncovered that receptor stimulation leads to cAMP production, not only from the plasma membrane but also from endosomes, once the receptor is internalized (60). This finding was confirmed with Nb37-GFP, that recognizes the alpha-5 helical domain of Gαs in the guanine nucleotide-free form that is an activation intermediate and is important for GPCR binding (61). At intra-cellular concentrations that do not inhibit Gαs activation, the Nb37-GFP biosensor is resident in the cytosol, and is recruited to the cell membrane upon ADRB2 activation. Even more importantly, this biosensor detects ligand-activated ADRB2 wherever it is located in the cell, hence providing precious informations on the sites of persistent ADRB2 cAMP signaling, such as the endosomes (**Figure 4**) (60). Similar observations were extended to the gastric inhibitory polypeptide receptor (GIPR), that also induces cAMP production once in the early endosomes (62). Single-molecule imaging with the Nb37-GFP biosensor has further uncovered that the alpha2A adrenergic receptor (ADRA2A) coupling to G protein is trapped to defined hot spots of interactions inside the plasma membrane, where signaling mainly occurs. These nanodomains are partially constituted by actin fibers, microtubules and clathrin-coated pits (63). Nanoclustering depends on the receptor conformational state, as demonstrated by single-molecule tracking of active and inactive conformers of ADRB2 bound to GFP-tagged Nb80, Nb37 or Nb60 biosensors in PC12 cells (64).

The NbSmo8 biosensor was the first intra-VHH biosensor directed against a class F GPCR. Similarly to anti-class A biosensors, it has been shown to bind the Smoothened homolog (SMO) only when activated by agonist binding (65).

Diverse ligands stabilizing discrete active states of the receptor induce its interaction with the conformational intra-VHH biosensors in a concentration-dependent manner, without the signal amplification that happens when measuring downstream signaling. Therefore, these intra-VHH biosensors may potentially provide a more reliable access to efficacy and potency of each ligand-receptor pair. Biosensors to monitor OPRM1, OPRD1, OPRK1 opioid receptors activity have been developed, either to monitor receptor activation state by different ligands, by quantifying biosensor interaction (42, 66), or to track activated receptor inside the cell (45, 66, 67). One of the latter ones has been developed to discriminate the side-effects of non-peptide clinically-relevant drugs *versus* the beneficial therapeutical effects of natural peptide ligands of OPRM1 (67, 68). This biosensor, derived from Nb33, is almost similar to Nb39, the intra-VHH that binds selectively to activated OPRM1 and OPRK1 described above (29, 42, 69). By total internal reflection fluorescence (TIRF) time-lapse experiments in neurons, the Nb33 biosensor reveals that met-enkephalin- or β-endorphin-activated OPRM1 or OPRD1 signals transiently from the plasma membrane, and then from receptor internalized in the endosomes. In contrast, non-peptide opioid drugs, such as morphine or etorphine, induce an additional, very early stimulation wave in the Golgi, that is independent of receptor trafficking (**Figure 3**). Similar results have been gathered when probing OPRK1 with the Nb39 conformational biosensor in cells stimulated with the SalA permeant drug or with Dynorphin, the OPRK1 natural ligand (45). These observations, emphasizing the notion of spatio-temporal bias, have wide implications for the development of neuro-modulatory drugs that generally penetrate the cell, hence are prone to illegitimate signaling from the Golgi. The latter might be involved in the well-known side-effects of opioids such as toxicity and addiction. With the use of Nb6B9, a high-affinity derivative of Nb80, agonist-activated D1A dopamine receptor (D1DR) or β1-adrenergic receptor (ADRB1) has also been observed at the Golgi apparatus in addition to the plasma membrane (70).

Intra-VHHs directed against the C-C chemokine receptor type 7 (CCR7) have been recently derived by random mutagenesis of CDR1 and CDR3 of Nb80 (71). Interaction of Nb1, Nb5 and Nb38 with CCR7 was tracked by bimolecular fluorescence complementation (BiFC) using split-YFP, regardless of the receptor activation state. They co-localized with CCR7 at the plasma membrane in resting conditions, and also in membrane ruffles and endosomes upon CCL19 stimulation, confirming that they do not recognize selective conformations of the receptor. Accordingly, they also modestly interfered with Gi binding to the receptor and Ca^2+^ response.

## Intra-VHHs to mimic transducer activity

Ligand binding induces conformational modifications of GPCRs that lead to the recruitment of intracellular transducers, among which G proteins and β-arrestins have been the most extensively studied. Intra-VHHs stabilizing G proteins or β-arrestins have served as tools to address the following questions, that are theoretically applicable to every GPCRs: i/the cryogenic electron microscopy (cryo-EM) structure of activated GPCR complexes with G proteins or β-arrestins, ii/the sub-cellular localization of signaling receptor, iii/the modulation of transducer activity and signaling outcomes, iv/understanding the relationships between β-arrestin conformational transitions and its activity, v/modulating the interactions of β-arrestins with their binding partners.

The structure of Gs-bound ADRB2 complexes has been resolved with the Nb35 as a structural chaperone (27). Because it recognizes the interface between Gαs Ras domain and Gβγ and prevents GTP dissociation when the trimer is bound to agonist-occupied receptor, Nb35 has helped solving the high resolution 3D structures of other active, Gs-coupled GPCRs, such as the thyrotropin receptor (TSHR) (72, 73), the lutropin-choriogonadotropic hormone receptor (LHCGR) (74) or the adenosine receptor A2a (ADORA2A) (75) among others, in active, transducer-bound conformation. In contrast to the paradigm establishing that β-arrestins binding precludes Gα binding on the receptor in order to desensitize it, it has been demonstrated by single-particle electron microscopy that Gαs and β-arrestin 1 can bind simultaneously the GPCRs that strongly interact with β-arrestins, such as the vasopressin V2 receptor (AVPR2) (76). In agreement, Nb35 has been used to stabilize the Gαβγ trimer within an activated ADRB2/AVPR2 chimeric receptor in complexation with both transducers (77) (**Figure 4**). The AVPR2 moiety represents the carboxyterminal region of the AVPR2 receptor that confers long-lasting interaction with β-arrestins, in contrast to ADRB2 that only transiently interacts with them (78). This study has profound biological impact because it revisits the role of β-arrestins as desensitizing agents presumably precluding Gs access to the receptor, by showing that β-arrestin and Gs binding is in fact not mutually exclusive. Gs occupies the core domain of the receptor, while β-arrestin 2 remains bound to the phosphorylated carboxy-terminal region of the receptor, in a so-called “tail” conformation. β-arrestins can also adopt a “core” conformation whereby they engage three structural elements, including the finger loop, into the core (transmembrane helices and intracellular loops) of the receptor for desensitization, their classically assigned role (77). Hence, by demonstrating that a GPCR coupled to β-arrestins can continue to signal through Gs, even if internalized, this “megaplex” hypothesis has provided a mechanistic clue on endosomal signaling, as presented above.

Because of its negative regulatory action on Gα through re-association, the Gβγ complex has raised interest as intra-VHH target. Therefore Nb5, that recognizes Gβ subtypes 1-4, has been developed (79). By suppressing Gβγ signaling, Nb5 inhibits Gβγ-regulated G protein-gated inward rectifier potassium (GIRK) channels signaling in medium spiny neurons of the striatum excited with DRD2- or CHRM4-mediated inhibitory post-synaptic current (IPSC). In response to apelin, Nb5 indirectly limits the inhibition of the Gi-coupled apelin receptor on forskolin-stimulated cAMP, by suppressing the inhibitory action of Gβγ on adenylate cyclase. Nb5 also inhibits downstream kinases such as Akt and Erk. But despite its inhibitory action on Gβγ functioning, this intra-VHH has no effect on GTP-bound Gα (**Figure 4**) (79).

Intracellular antibodies against β-arrestins exhibiting interesting biological properties have also been characterized, although the first ones were not VHHs but scFv or Fabs. For example, scFv5 disrupted the interaction of β-arrestins with clathrin and inhibited the endocytosis of 8 different GPCRs, suggesting a generic value of this scFv as a tool to assess GPCR internalization (80). In addition, a Fab, Fab30, that recognized an active “tail” conformation of β-arrestin 1 when bound to a phosphorylated carboxyterminal region of V2R (81), was isolated. From Fab30, an intra-cellular scFv, named Ib30 by the authors, has been derived as a biosensor to monitor β-arrestin 1 recruitment to chimeric as well as GPCRs with a native carboxyterminal region, and then to track β-arrestin trafficking inside the cell (82). Importantly, Ib30 confers a gain-of-function in β-arrestin 1 conformation, since it restores its translocation to endosomes and its ability to stimulate ERK MAP kinases, otherwise impaired upon agonist-stimulation of the AVPR2 T360A phosphorylation mutant (83). Structural analysis and molecular dynamics indicate that only Lys11 of β-arrestin 1 phosphate sensor remains engaged by ionic interactions with the phosphorylated receptor and not Lys 294 in the polar core, nor Arg25, that are also involved in fully activated conformation of β-arrestin 1. This analysis explains that, when bound to the AVPR2 T360A mutant, β-arrestin 1 adopts a partially active conformation that can be allosterically modified to accommodate β2-adaptin interaction and subsequent internalization. The use of Ib30 as a biosensor to monitor β-arrestin 1 conformational changes has been extended to other GPCRs (82, 84, 85).

Finally, *bona fide* camelid anti-β-arrestin 1 intra-VHHs came out, and one of them, Nb32, has been very useful to investigate the functional outcomes of β-arrestin 1 binding to the GPCR core and/or to the tail (**Figure 4**) (86). This antibody binds β-arrestin 1 only when associated to a receptor. When comparing a β-arrestin 1 deleted of its finger loop, hence devoid of its ability to bind the core domain of the receptor, and the Nb32-stabilized β-arrestin 1, it appeared that β-arrestin 1 binding to the tail predominantly drives the receptor to internalization and promotes signaling, but is not able to promote G protein desensitization, as expected from the megaplex model (76, 77).

So far, the anti-β-arrestin antibody fragments that have been described allosterically modulate β-arrestin/GPCR interaction by stabilizing selective receptor conformations. In the future, it will be of great interest to stabilize selective, fully or partially engaged conformations of β-arrestins instead. In this way, it should be possible to control independently receptor-mediated signaling and trafficking. Beyond β-arrestin/GPCR interaction, only scFv5 has been shown to impair the interaction of β-arrestin 2 with one of its non-receptor interaction partner (80). This finding opens the promising possibility of interfering with β-arrestin signaling partners. This is of paramount interest because β-arrestins undergo interactions with hundreds of signaling proteins, including at least 17% of direct interactions, leading to diversified, yet essential biological responses (87–89). Hence, intra-VHHs directed against precise interfaces of β-arrestins might help to decipher the specificity of their signaling outputs.

## Methodological limitations and opportunities

**Figure 5:**
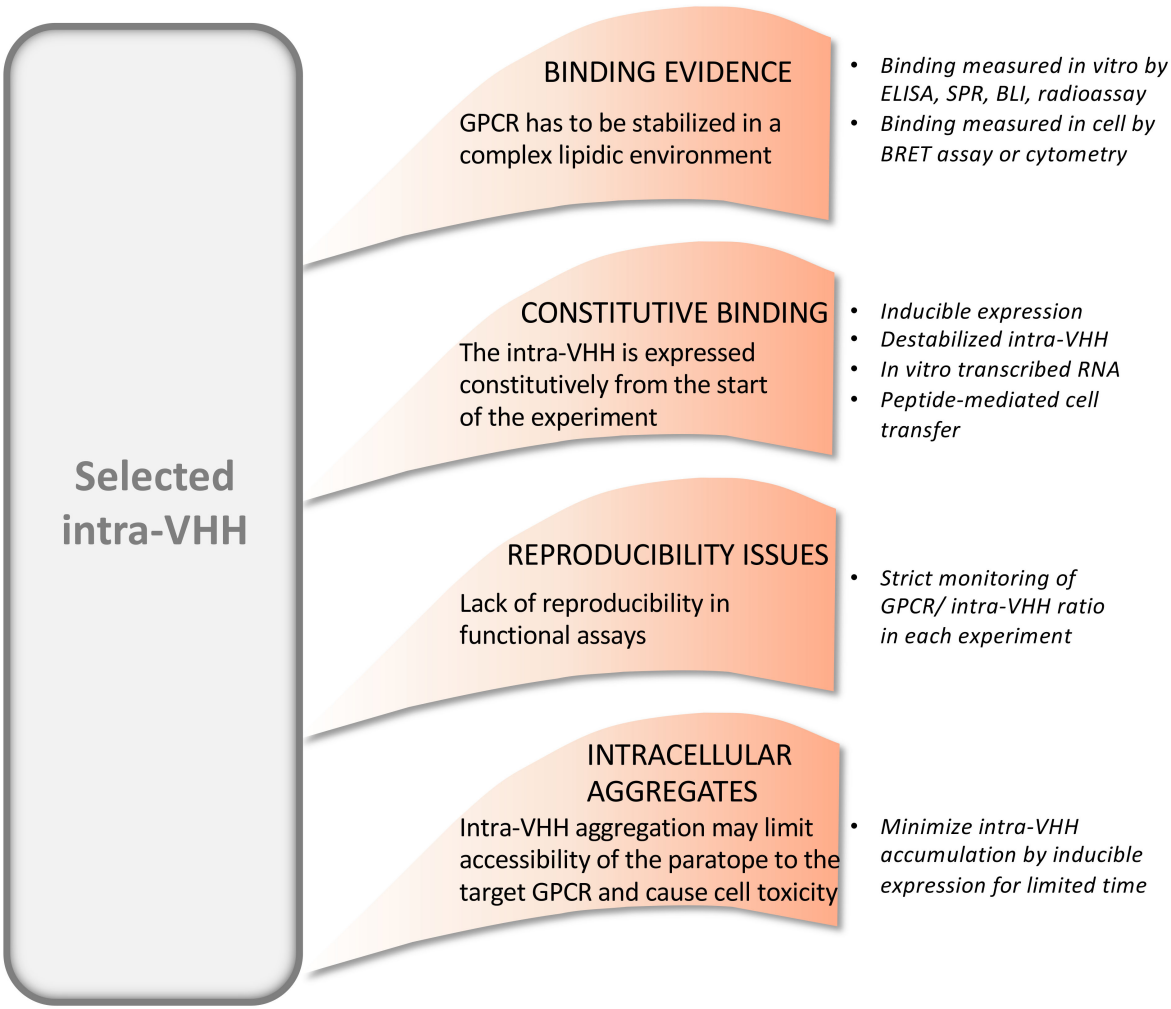
Examples of methodological challenges associated to functional studies on intra-VHHs that modulate GPCR signaling, and the existing solutions.

Methodological hurdles that linger the development of VHHs against GPCR, such as the selection of the antigen (90), the advantages of synthetic libraries versus libraries from immunized animal (40, 91, 92), the comparison of display methods (5, 38, 40) have been previously reported. Here, we focus on the specificities that make anti-GPCR intra-VHH expression inside the cell and characterization particularly challenging (**Figure 5**).

One is that the anti-GPCR intra-VHHs isolated so far are generally conformational antibodies that recognize non-linear epitopes within the inner core domain of the receptor, as shown for Nb80. However, the intracellular loops, especially ICL3, and the carboxyterminal region of GPCR are intrinsically disordered, which may be a disadvantage to select specific, high-affinity VHHs.

The key parameter to be monitored when isolating an antibody against a selected target is direct antibody/antigen binding. But *in vitro* binding by enzyme-linked immunosorbent assay (ELISA), surface plasmon resonance (SPR) or interferometry can be a challenging task when the target is a GPCR that cannot be easily produced and purified in native, active conformation. Assaying intra-VHH-GPCR binding in cell can be an alternative by using BRET (42–44) or the developing Nanobit complementation technology (83, 93). Cytometry is also an efficient means (39), but low level of endogenous or exogenously expressed recombinant receptor and limited intracellular affinity of the VHH may lead to signal below the detection threshold. In addition, *in vitro* affinity may not be linearly correlated to efficacy in the reducing milieu of the cytoplasm, as shown for the anti-ADRB2 Nb.c203, which exhibits moderate affinity maturation in yeast when compared to the other intra-VHHs selected, but was the most efficient to decrease the Emax of adrenaline to produce cAMP. Similarly, the folding of a VHH produced in bacteria and expressed from inside the cell may be different, which adds another level of variability when assessing affinity. These observations underscore the necessity to test several intra-VHHs to obtain a maximum effect on receptor signaling.

Since the VHH is generally expressed inside the cell from a transfected plasmid, it can be difficult to accurately control its quantity from the beginning of the experiment. If it recognizes inactive conformations of the receptor, it would be bound to the GPCR prior to agonist stimulation. Therefore, if its affinity is high enough, it should prevent transducer binding and lead to functional inhibition of downstream signaling. But if it is not, then it should be displaced by the ligand-activated transducers, without any detectable functional outcome, which may lead to bewildering conclusions. One alternative is to express the VHH intracellularly from an inducible promoter, as previously done with anti-US28 VUN13 intra-VHH (44), which also minimizes off-target reactions. Quite interestingly, destabilizing mutations of an intra-VHH that are silent only in the presence of the specific antigen have been reported (94). These mutations constitutively drive the intra-VHH to the proteasome-degrading pathway, unless the antibody is bound to its cognate antigen. Hence, an intra-VHH may be permanently degraded until its cognate GPCR target adopts the suitable active conformation upon ligand binding. This work is of broad potential applications because the destabilizing mutations are present in the structurally conserved framework regions of the antibody. In addition, several optically-controlled intra-VHHs have been engineered [reviewed in (95)]. Additional endeavors have to be pursued, based on the introduction of the antibody with a cell-penetrating peptide (96), or the transfection of *in vitro*-transcribed RNA, to detect the intra-VHH as soon as 3 hours after introduction (97).

The fact that it is very difficult to date to control precisely the expression level of an intra-VHH severely compromises reproducibility between independent experiments, especially because the stoichiometric ratio between the receptor and the VHH, by altering ligand binding affinity, indirectly alters the efficacy to stimulate a given functional readout.

In several instance, the question remains as whether conformational intra-VHHs that have been reported are really specific of one receptor, or if they recognize active/inactive conformations that are common to several receptors of the same family (ex, the opioid receptors, adrenergic receptors, etc) or even receptors from distinct classes (ex, class A GPCR vs class F). As indicated above, Nb6B9 recognizes D1DR in addition to ADRB1 and ADRB2 (70). For class A GPCR, cross-reactivity could reflect the relative conformational conservation of their intracellular half, that accommodates to a limited number of G proteins and β-arrestins (98, 99).

## Therapeutic opportunities

The explosion of structures of GPCRs in active conformations since 2011 has paved the way for the rational, structure-based design of new drugs of potential therapeutic interest.

**Figure 6:**
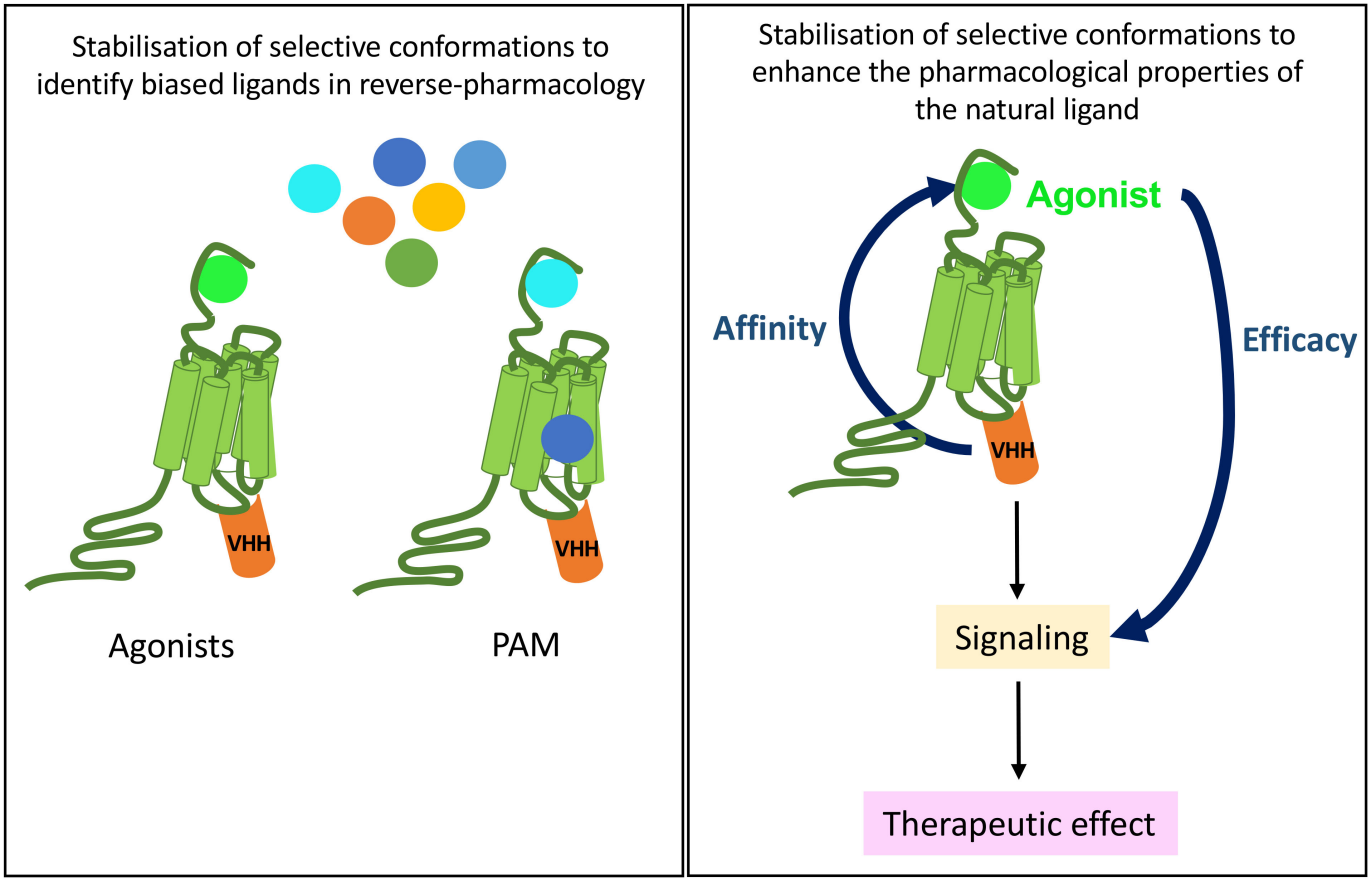
Potential therapeutical applications of anti-GPCR intra-VHHs. A GPCR immobilized by an intra-VHH in an active conformation of biological interest may serve as a target for drug discovery of new biased ligands, be they allosteric or orthosteric (left). *In vivo*, provided that intra-VHH may be vectorized into the appropriate target cell, they could allosterically modulate the pharmacological properties of the endogenous ligand, which, for example, would respect the endocrine physiological rhythms and control.

In drug discovery, one moonshot is to enhance binding affinity of biased agonists of therapeutical interest because, by stimulating various signaling pathways with distinct efficacy when compared to the natural agonist, they are expected to lead to less side-effects (**Figure 6**). If a GPCR is stabilized in a defined active conformation with an intra-VHH, then the affinity of not only its natural ligand will increase but also any other biased ligand with interesting signaling properties. This is the purpose of reverse pharmacology, where a receptor is locked in an active conformation with an intra-VHH, to screen for (allosteric/biased) ligands endowed with new pharmacological properties when compared to the reference agonist. A proof-of-concept of reverse pharmacology has been successfully applied to ADRB2 fused to the transducer-mimicking Nb80 and to the OPRM1-Nb33 fusion, in a fragment-based screening of 1000 small molecules (38, 100, 101). This comparative fragment screen has led to the pharmacological classification of multiple novel ligands as agonists, antagonists, inverse agonists, etc, on the basis of their efficacy to stimulate readouts of interest (100). Fragment-derived compounds binding the orthosteric site of ADRB2-Nb80 fusion with affinity in the nM range were selected, and they discriminate accurately the active and basal states of the receptor. Hence, this strategy raises hopes to the selection and characterization of ligands selective of a given receptor conformation of interest, in a cell-free system.

In the ADRB2-Nb80 complex, a β hairpin conformation of the VHH CDR3 engages interactions with the receptor overlapping the Gαs binding site (24). This prompted research groups to launch rational design programs using CDR3 β hairpin peptidomimetics to stabilize active conformations of ADRB2 for ligand screening (102). Two of these peptidomimetics inhibited cAMP production, as expected from competition with endogenous Gαs. Nevertheless, the effect was modest when compared to Nb80 inhibitory effect. These attempts still need more efforts for success, probably because peptidomimetics for ADRB2 fail to fully stabilize active conformations of the receptor (103, 104).

Importantly, intra-VHHs have the unique potential to isolate GPCR agonists, not only as small chemical compounds, but also in the VHH format, recognizing the extracellular regions of a receptor. As indicated above, VHHs with agonistic have only been described for APLNR and GRM2. Hence, intra-VHHs may increase the discovery of new agonistic VHHs to GPCR of clinical relevance.

Another application of intra-VHHs in drug discovery for GPCR can be foreseen through the example of atypical chemokine receptor 3 (ACKR3), that is naturally biased towards GRK and β-arrestin signaling, because of the absence of a kink of TM4 that leads to a clash of ICL2 and Gα (105, 106). Hence, it is conceivable that an intra-VHH stabilizing this compact conformation of the Gα-binding cleft would increase the probability to isolate new biased ligands of this receptor.

The exquisite properties of anti-GPCR VHHs, such as specificity, affinity, short serum half-life, open exciting opportunities for future therapeutics. Obviously, these properties are shared with intra-VHHs. However, in the latter case, even more critically than for *in vitro* applications, a major issue to be overcome is their functional delivery into target cells *in vivo*, although some progress are being made, as reviewed in (107). This is why, to date, no example of intracellularly delivered VHH has been reported.

## Conclusions

Intra-VHHs have widely proven their utility as chaperones for structural studies of GPCR by X-ray crystallography or cryo-EM. In contrast, the characterization of anti-GPCR intra-VHHs with functional properties inside the cell is still poorly developed. So far, the functional impact of only very few intra-VHHs on GPCR signaling activity has been characterized, when considering that only ADRB2, OPRK1 and US28 and, to a lesser extent, HTR2A, have been targeted to date (**Table 1**).

**Table 1:** Summary of intra-VHHs modulating GPCR signaling and/or on trafficking cited in this paper.

Intra-VHH	Target	Signaling	Reference
Nb80	Active ADRB2	Gαs mimic Disrupts the ionic lock between TM3 and TM6. Decreases cAMP response (Gαs competition) and β-arrestin recruitment	(24, 39)
Nb80-GFP	Active ADRB2	Probes receptor activity from the plasma membrane and from the endosomes	(60)
Nb6B9	Active ADRB2, ADRB1, D1DR	Nb80 high affinity derivative. Detects active D1DR and ADRB1 at the plasma membrane and in the Golgi	(70)
Nb60	Inactive ADRB2	Prevents cAMP response and β-arrestin recruitment	(39)
Nb71	Partially active ADRB2	Partial outward shift of TM6. Decreases cAMP response. Inhibits receptor phosphorylation by GRKs and β- arrestin recruitment	(30, 39)
Nbc.200	ADRB2 stimulated with adrenalin	Decreases cAMP response	(40)
Nb6	Inactive OPRK1	Binds ICL3. Displaced by Gq recruitment	(42)
Nb39	Active OPRK1	No effect on Gi binding	(42, 45)
Nb39-GFP	Active OPRK1	Detects signaling from plasma membrane and from endosomes with dynorphin. and also from Golgi if non-peptide opioid	(67)
VUN103	Constitutively active US28	Binds ICL2 and ICL3. Displaces Gq and β-arrestin 2. Impairs IP3 accumulation, NFAT, NF-kB and STAT3 activity	(44)
Nb7	Ligand-activated US28	Binds ICL2 and ICL3. Neutral to constitutive activity	(44)
VGS-Nb2	Active HTR2A	PAM even in the absence of Gq. Stimulates SRE activation, decreases β-arrestin 2 recruitment	(43)
NbSmo8-GFP	Active SMO	Detects active SMO at the plasma membrane	(65)
Nb33-GFP	Active OPRM1. OPRD1	Detects signaling from plasma membrane and from endosomes with met-Enk. and also from Golgi if non-peptide opioid	(67)
Nbl	Inactive and active CCR7	Slightly interferes with Gi binding. Detects CCR7 at the plasma membrane in basal conditions and also in membrane ruffles and vesicules upon CCL19 stimulation	(71)
Nb38	Inactive and active CCR7	Inhibits Gi binding. Detects CCR7 at the plasma membrane in basal conditions and also in membrane ruffles and vesicles upon CCL19 stimulation	(71)
Nb37-GFP	Alpha-5 helical domain of nucleotide-free Gαs	Detects active Gαs at the plasma membrane and at the endosomes (ADRB2 and GIPR). Identifies hot spots of Gαs-dependent signaling in the plasma membrane (ADRA2).	(60, 62, 63)
Nb35	GPCR bound Gαs and Gβγ interface	Prevents Gαs and Gβγ dissociation upon activation. Stabilizes the megaplex	(27, 76, 77)
Nb5	Gβ subtypes 1-4	Suppresses Gβγ signaling, Akt and ERK signaling	(79)
Nb32	Activated β-arrestin 1	Stabilises β-arrestin binding to the GPCR core and promotes desensitization	(77, 86)

Only the targets for which a signaling outcome has been measured are indicated. Below the dark line, the intra-VHHs recognizing G proteins or β-arrestins are also indicated.

One clear bottleneck is the difficulty to properly characterize their pharmacological properties in a complex cell system and to assess specificity despite likely intracellular off-targets. Screening for drugs that bind to conformationally stabilized binding pockets within GPCR might lead to more selective and efficient therapeutics. Intra-VHHs indirectly mimic the effect of biased agonists, from the intracellular side.

In the future, exploration of the functionality of intra-VHHs on a given GPCR will need to be systematically coupled to resolution of its structure. Only in this condition will it be possible to approach the issue of conformation/activity relationship, to correlate a (biased) ligand to a receptor conformation or ensemble of conformations, to a signaling pathway, or even network, and ultimately, integrated biological response. Still, one caveat is that the conformations that are co-crystallized do not necessarily predict accurately the conformations that are preferentially stabilized in a complex cell system.

## Author contributions

All authors significantly contributed to conceptualization and writing of the manuscript, and to the design of the figures. All authors contributed to the article and approved the submitted version.

## Funding

PR is funded by a joint fellowship from Région Centre Val de Loire and INRAE PHASE Department; CG and VJ are funded by the SELMAT grant of Région Centre Val de Loire ARD2020 Biomédicaments Program. CG is co-funded by the INTACT grant from Région Centre Val de Loire; GB and ER are funded by INRAE, FJ-A and PC are funded by the CNRS. This publication was funded with support from the French National Research Agency under the program “Investissements d’avenir” Grant Agreement LabEx MabImprove: ANR-10-LABX-53; “ARD2020 Biomédicaments” and “APR-IR INTACT” grants from Région Centre Val de Loire; Bill & Melinda Gates Foundation.

## Acknowledgments

The authors would like to acknowledge Région Centre Val de Loire and the MAbImprove Labex for their constant support of our research on antibody fragments.

## Conflict of interest

The authors declare that the research was conducted in the absence of any commercial or financial relationships that could be considered as a potential conflict of interest.

## Publisher’s note

All claims expressed in this article are solely those of the authors and do not necessarily represent those of their affiliated organizations, or those of the publisher, the editors and the reviewers. Any product that may be evaluated in this article, or claim that may be made by its manufacturer, is not guaranteed or endorsed by the publisher.

## Abbreviations

GPCR, G protein-coupled receptor; VHH, V_H_ of heavy-chain only antibodies; Nb, nanobody; sdAb single-domain antibody; CDR, complementarity-determining region.
